# Factors associated with early implant discontinuation among women receiving implant services from community healthcare providers (CPs/PPMVs) in Nigeria: an analysis of routine service delivery data

**DOI:** 10.3389/fgwh.2026.1827336

**Published:** 2026-07-20

**Authors:** Sikiru Baruwa, Kolawole Oni, Toyin O. Akomolafe, Emeka Emmanuel Okafor, Osimhen Ubuane, Rodio Diallo, Michael Alagbile, Jennifer Ladokun

**Affiliations:** 1 Research Impact, Population Council, Abuja, Nigeria; 2 IntegratE Project, Society for Family Health, Abuja, Nigeria; 3 Family Planning, Country Impact Team & Technical Support, Gates Foundation, Seattle, WA, United States

**Keywords:** community pharmacists (CPs), family planning, implant discontinuation, long-acting reversible contraceptives (LARCs), Nigeria, patent and proprietary medicine vendors (PPMVs)

## Abstract

**Background:**

Contraceptive implants are highly effective long-acting reversible contraceptives (LARCs), yet early discontinuation remains a major challenge globally and in Nigeria, undermining family planning (FP) goals. Community Pharmacists (CPs) and Patent and Proprietary Medicine Vendors (PPMVs) have been trained through the IntegratE Project, to expand access to implant, but evidence on discontinuation patterns among their clients is limited.

**Methods:**

This retrospective descriptive study analyzed 874 routine service delivery records of women aged 15–49 years who sought implant removal services from trained CPs and Tier 2/3 PPMVs between July 2023 and June 2025 across four Nigerian states (Enugu, Kaduna, Kano, and Lagos). Early discontinuation was defined as removal within two years of insertion. Descriptive statistics summarized socio-demographic and service-related characteristics, while bivariate and multivariate logistic regression identified predictors of early removal.

**Results:**

Overall, 28.4% (*n* = 248) of implant users who discontinued discontinued early, with similar proportions for Implanon (29.5%) and Jadelle (25.7%%). More than half (57.6%) of early discontinuers removed their implants within 12 months of insertion. Side effects (38.9%) and desire for pregnancy (37.2%) were the most reported reasons for discontinuation whether early or not, while partner disapproval accounted for 15.4%. Adjusted analysis showed women aged 35–39 years were less likely to discontinue early compared with those aged 25–29 years [Adjusted odds ratio (AOR) = 0.50; 95% confidence interval (CI): 0.31–0.80; *p* = 0.004]. Geographic variation was noted, with lower odds in Enugu (AOR = 0.53; 95% CI: 0.30–0.91; *p* = 0.02). Side effects (AOR = 3.01; 95% CI: 1.89–4.78; *p* < 0.001) and partner disapproval (AOR = 2.31; 95% CI: 1.25–4.26; *p* = 0.007) were significant predictors.

**Conclusion:**

Almost one-third of discontinuers served by CPs and PPMVs did so early that is at less than 24 months, primarily driven by side effects and partner opposition. Strengthening counselling, managing side-effects, and engaging male partners are critical to improving continuation and advancing Nigeria's FP goals.

## Introduction

Globally, family planning (FP) is recognized as a cornerstone of reproductive health and a critical strategy for reducing maternal and child mortality, preventing unintended pregnancies, and advancing women's empowerment (1). Among modern contraceptive methods, long-acting reversible contraceptives (LARCs), particularly implants, have gained increasing attention due to their high efficacy, safety, and convenience. Contraceptive implants provide protection for three to five years, with effectiveness exceeding 99% (2, 3). Their popularity continues to rise because they are user-independent, discreet, reversible, and suitable for women across different age groups, including adolescents and those wishing to space or limit births (4).

Across Africa, the past decade has witnessed substantial expansion in access to modern contraceptives. This progress has been driven largely by global partnerships such as Family Planning 2020 (FP2020) and commitments from the International Conference on Population and Development (ICPD) (5). Implants have become a key part of this expansion, with several countries incorporating them into national FP strategies (6). Despite this growth, discontinuation rates remain high. Evidence shows that 15%–40% of women discontinue implant use within the first year, this is often driven by side effects, desire for pregnancy, or partner opposition (7–9). Such early discontinuation undermines efforts to reduce maternal mortality, prevent unsafe abortions, and achieve Sustainable Development Goals (SDGs) 3.1 and 3.7, which emphasize universal access to sexual and reproductive health services (10–12). In West Africa, contraceptive discontinuation is particularly concerning. Implants account for an increasing share of the contraceptive method mix, and lower discontinuation rates compared to other methods (13).

Nigeria, the most populous country in Africa, reflects these changes vividly, with implant contraceptive prevalence rate increasing from 3% to 6% and implant discontinuation rate of 18% (14). Despite being a priority country for global FP initiatives, modern contraceptive prevalence remains low (15%), while unmet need among married women is high (21%) (14–16). Implants have become an important component of Nigeria's contraceptive method mix, helping to address the wide gap between contraceptive demand and supply (17–19). Yet, early discontinuation persists and increases the risk of unintended pregnancies, with serious implications for maternal and child health (19, 20).

Community-based providers play an essential role in addressing these gaps. Community pharmacists (CPs) and patent and proprietary medicine vendors (PPMVs) are often the first point of contact for health services, especially in underserved and hard-to-reach communities (21). They are highly accessible and trusted, serving as critical links between communities and the formal health system. Through initiatives such as the IntegratE Project, CPs and PPMVs have been trained to provide family planning counselling, implant insertions and removals, and referrals in states such as Lagos, Kaduna, Kano, and Enugu (22). Evidence suggests that engaging and training community providers expands access to FP services and improves women's method choices (17, 23). Studies indicate that the type of provider who inserts the implant is associated with the likelihood of early discontinuation (9). However, there is limited empirical research on how women's experiences with CPs and PPMVs influence implant continuation or discontinuation.

This study therefore examines the factors influencing early implant discontinuation among women accessing services from trained CPs and PPMVs in Enugu, Kaduna, Kano, and Lagos States, Nigeria. By exploring the current practice in Implanon discontinuation at the community health providers level, the study seeks to inform policies and interventions aimed at improving counselling quality, strengthening community-based service delivery, and enhancing contraceptive continuation. Ultimately, the findings will contribute to Nigeria's efforts to expand access to high-quality FP services and achieve national and global reproductive health targets.

## Methods

### Intervention

Through the participation of private sector providers, CPs and PPMVs, the IntegratE project sought to expand access to contraceptive methods in underprivileged and underserved communities across 11 states in Nigeria. The Gates Foundation-funded project collaborated with the government and other relevant parties to give CPs and PPMVs standardized training in family planning counseling and method options. In order to fulfill Nigeria's commitment to a modern contraceptive prevalence rate (mCPR) of 27 percent among all women by 2024, the project expanded access to modern contraception by increasing the number of service delivery points offering FP services and incorporating PPMVs in task shifting policy implementation (24). IntegratE aimed to show that CPs and health-trained PPMVs (Tier 2 and 3 PPMVs) can offer FP services, including implants and injectables, of a quality comparable to what is offered in the public sector with the right training and supportive supervision.

The Pharmacy Council of Nigeria (PCN), a regulatory body, and the Project, collaborated to pilot a three-tier accreditation system for PPMVs based on their qualifications in healthcare (25). Based on their health qualifications, PPMVs were divided into three tiers and, depending on their cadre, trained in counseling techniques, family planning methods: use, efficacy of methods, and side effects (25). The three tiers are Tier 1 without a health qualification and Tier 2 and Tier 3 having health qualifications. CPs are not part of the accreditation system but they have received similar training to PPMVs with health qualifications. All PPMVs and CPs were trained in injectables and implants, with the exception of non-health trained PPMVs, who are classified as Tier 1 (25). 2,000 CPs and health trained PPMVs (Tier 2 and Tier 3) from Kano, Kaduna, Enugu and Lagos states received training on implants and injectables from the IntegratE project between January 2022 and September 2023. A detailed description of the IntegratE Project, the three-tier accreditation system, the type of training, and the tiered PPMVs can be found elsewhere (25).

### Study design and setting

This study employed a retrospective descriptive design, drawing on routine service delivery data. The analysis focused on contraceptive implant removals documented by community-based health providers trained under the IntegratE 2.0 Project. The study was conducted in four Nigerian states: Enugu, Kaduna, Kano, and Lagos. These states were selected because of their diverse sociocultural and health system contexts, representing both urban and rural populations, and reflecting regional differences in FP demand, service delivery, and cultural acceptance of contraceptives. Lagos, Nigeria's commercial hub, is urbanized and diverse, with relatively higher access to health services. Kaduna and Kano, in northern Nigeria, are shaped by strong cultural and religious norms that influence reproductive behavior, male partner involvement, and FP acceptance. Enugu, in southeastern Nigeria, represents a different sociocultural context, where health-seeking behavior and community norms vary significantly from those in the north (14). Understanding these differences is critical for identifying the factors that drive early implant discontinuation across different cultural and geographical settings.

### Data source, population and eligibility criteria

Data were extracted from routine service records on contraceptive implant removals between July 2023 and June 2025. The study population comprised women of reproductive age (15–49 years) who sought removal of contraceptive implants from trained CPs and Tier 2 and Tier 3 PPMVs during the review period in Enugu, Kaduna, Kano, and Lagos States. A total of 874 client removal records which met the inclusion criteria (clients dates of implant removal) were identified and extracted from the family planning daily register and client cards available at CPs and PPMVs' premises and were analysed.

All records of women between the ages of 15–49 years who were using implants and came to the CPs and PPMVs for removal during the period under review (July 2023 and June 2025) were included in the study. Women who came for implant insertion at CPs and PPMVs' premises but removed elsewhere or came for other family planning services were excluded from the study.

### Sample size determination and sampling procedure

The study sample size was determined by the number of client records that met the inclusion criteria during the review period. All service records of women of reproductive age (15–49 years) who sought removal of contraceptive implants from trained community-based providers between July 2023 and June 2025 in Enugu, Kaduna, Kano, and Lagos States were reviewed. A total of 874 client records of clients who have removed implants were identified and included in the analysis. No formal sample size calculation was conducted, as this was a retrospective review of routine service data. The final sample size therefore represents the entire population of eligible implant removal cases documented by trained Community Pharmacists (CPs) and Tier 2 and Tier 3 Patent and Proprietary Medicine Vendors (PPMVs) within the study period and states.

### Study variables

The *dependent variable*, early implant discontinuation is defined as the removal of implant before completing 2 years since its insertion. It was dichotomously categorized as early contraceptive implant removal (yes, if ≤ 24 months) or not (no, if > 24 months) (26).

The *independent variables* considered were socio-demographic variables of participant characteristics such as age, residence, religion and the contraceptive related factors. The contraceptive related factors include side effects, desire to get pregnant and partner decision and were dichotomized into yes or no.

### Data quality control

Data quality assurance procedures were applied at multiple stages to ensure the accuracy and completeness of the records. Service data were first collated from routine FP register maintained by trained Community Pharmacists (CPs) and Tier 2 and 3 PPMVs across the four study states. Records were checked for completeness, internal consistency, and logical accuracy (e.g., ensuring removal dates followed insertion dates). Duplicate entries were identified and removed, while incomplete records with missing key variables (such as client age or reason for removal) were excluded. Data cleaning and validation were conducted in collaboration with project monitoring and evaluation (M&E) teams to ensure adherence to national family planning reporting standards.

### Ethical approval

This study was a retrospective, secondary analysis of routine data. Ethical approval for the IntegratE project was obtained from Population Council's IRB (878). Because the study was conducted through a review of family planning service records, informed consent was not required. Data is de-identified and personal identifiers (names, IDs, addresses, etc.) were not included in the data collection format.

### Data processing and analysis

Data were entered into Microsoft Excel and exported to SPSS version 26.0 for analysis. Prior to analysis, the dataset was cleaned to remove duplicates, resolve inconsistencies, and exclude incomplete records lacking key variables such as age, type of implant removed, or reason for removal. Descriptive statistics were used to summarize client socio-demographic characteristics, provider type, implant type, duration of use, and reasons for removal. Categorical variables were presented as frequencies and percentages, while continuous variables such as client age and duration of implant use were summarized using means and standard deviations. Graphical presentations (e.g., bar charts and frequency tables) were used to display key findings.

The primary outcome variable was early implant discontinuation, defined as removal of an implant before the recommended effective duration (26). To examine associations, bivariate logistic regression analysis was conducted between early implant discontinuation and selected explanatory variables, including client age group, state (Enugu, Kaduna, Kano, Lagos), type of implant removed, duration of use, provider type (Community Pharmacist vs. Tier 2 or 3 PPMV), and reasons for removal (e.g., side effects, desire for pregnancy, partner influence). Odds ratios (ORs) with 95% confidence intervals (CIs) were calculated, and statistical significance was set at *p* < 0.05. Variables with *p* < 0.20 in bivariate analysis were further entered into a multivariate logistic regression model to adjust for potential confounders such as age, state, and provider type. Adjusted odds ratios (AORs) with 95% CIs were reported to identify independent predictors of early implant discontinuation.

## Results

### Demographic information of study participants

Table 1 presents the demographic characteristics of the participants. The study included 874 women visited trained community providers in the selected states, The age of respondents ranged from 15 to 49 years, with the mean [ ± standard deviation (SD)] age of 31.8 ± 7.8 years. Most participants were in their prime reproductive years (25–34 years), accounting for 46.1% of the women, while only 16 (1.8%) were adolescents (15–19 years) and 74 (8.5%) were aged 45 years or older. In addition, the majority lived in semi-urban 371 (42.4%) and urban 365 (41.8%) areas, while only 138 (15.8%) resided in rural communities.

**Table 1:** Characteristics of women.

Variables	Category	Frequency	Percent
Age of women	15–19	16	1.8
	20–24	150	17.2
	25–29	211	24.1
	30–34	192	22.0
	35–39	145	16.6
	40–44	86	9.8
	≥45	74	8.5
	Mean ± SD	31.75 ± 7.77	—
State of residence	Enugu	61	7.0
	Kaduna	389	44.5
	Kano	195	22.3
	Lagos	229	26.2
Residence type	Rural	138	15.8
	Semi-urban	371	42.4
	Urban	365	41.8

### Implant type and service delivery points

Table 2 shows the implant type and service delivery points of implant insertion. Of the total implants removed, 606 (69.3%) were Implanon, while 268 (30.7%) were Jadelle. More than half of the implants were inserted at Primary Health Care (PHC) facilities 456 (52.2%), followed by Patent and Proprietary Medicine Vendors (PPMVs) 346 (39.6%), While smaller proportions were inserted at private hospitals 27 (3.1%), and pharmacies 9 (1.0%). For implant removals, nearly all were removal by PPMVs 863 (98.7%), with only 11 (1.3%) removals recorded at pharmacies.

**Table 2:** Implant type and service delivery points.

Variables	Category	Frequency	Percent
Implant type	Implanon	606	69.3
	Jadelle	268	30.7
		
Place of insertion	Pharmacy	9	1.0
	PHC	456	52.2
	PPMV	346	39.6
	Private hospital	27	3.1
	Secondary hospital	36	4.1
		
Place of removal	Pharmacy	11	1.3
	PPMV	863	98.7

### The magnitude of early implanon discontinuation

Table 3 presents the implant discontinuation and duration of use by implant type among study participants**.** The magnitude of early implant discontinuation is presented in Table 3. Overall, 248 women 28.4% discontinued their implants early out of 874 implant users who discontinued. Early discontinuation proportions were nearly identical between implant types: 29.5% among Implanon users and 25.7%; among Jadelle users. Among Implanon early discontinuers, 37 (20.7%) discontinued between 1 and 6 months, 66 (36.8%) within 7–12 months, 52 (29.1%) within 13–18 months, and 24 (13.4%) between 19 and 24 months of insertion. For Jadelle early discontinuers, 14 (20.3%) discontinued within 1- 6 months, 23 (33.3%) within 7–12 months, 18 (26.1%) within 13–18 months, and 14 (20.3%) between 19 and 24 months.

**Table 3:** Early implant discontinuation and duration of use by implant type among study participants.

Implant type	Early discontinuation		Total	Duration of use (Yes only)
	No *n* (%)	Yes *n* (%)	n	n (%)
Implanon	427 (70.5)	179 (29.5)	606	1–6 Months: 37 (20.7) 7–12 Months: 66 (36.8) 13–18 Months: 52 (29.1) 19–24 Months: 24 (13.4)
Jadelle	199 (74.3)	69 (25.7)	268	1–6 Months: 14 (20.3) 7–12 Months: 23 (33.3) 13–18 Months: 18 (26.1) 19–24 Months: 14 (20.3)
Total	626 (71.6)	248 (28.4)	874	1–6 Months: 51 (20.6) 7–12 Months: 89 (35.9) 13–18 Months: 70 (28.2) 19–24 Months: 38 (15.3)

**Figure 1:**
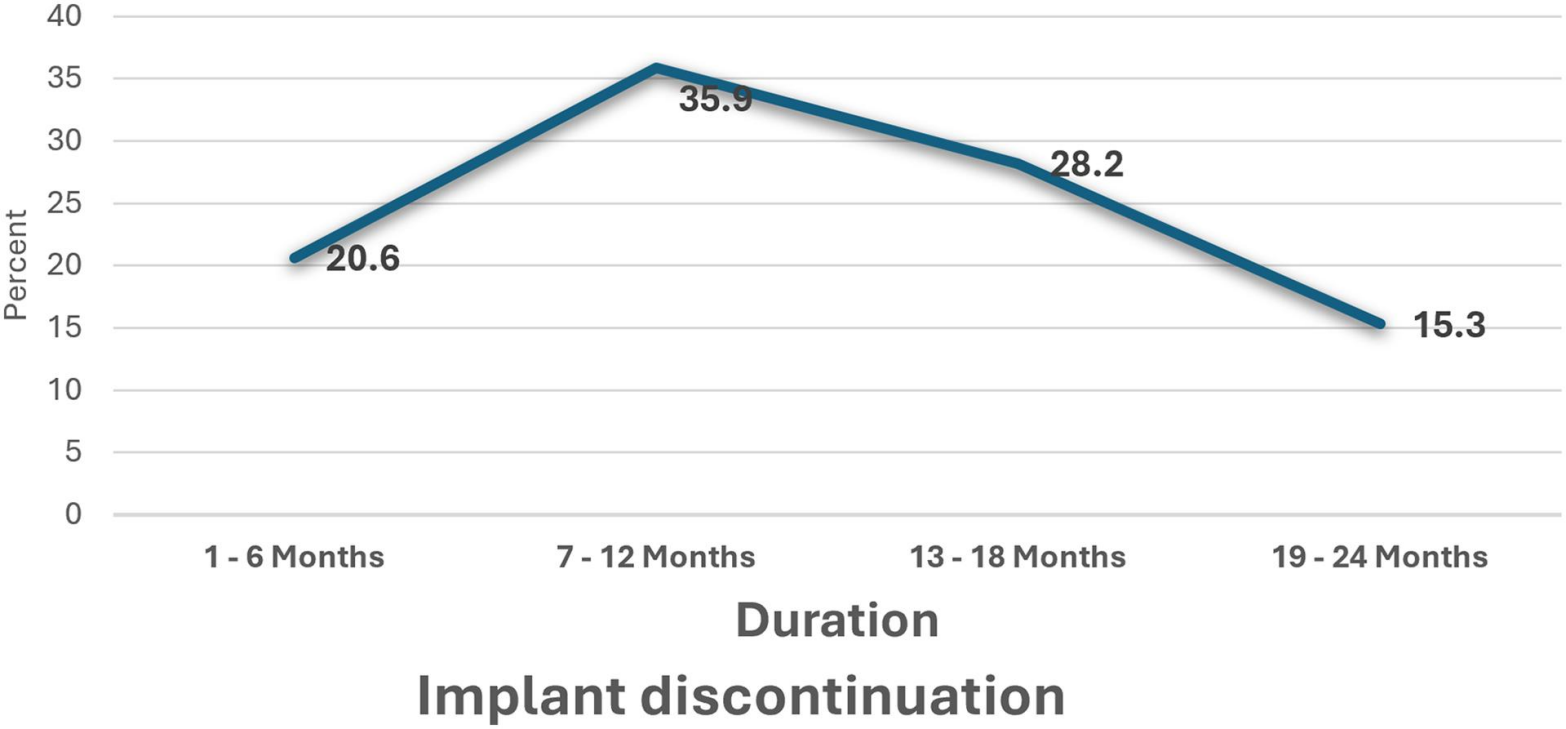
Duration at discontinuation by implant type.

**Figure 2:**
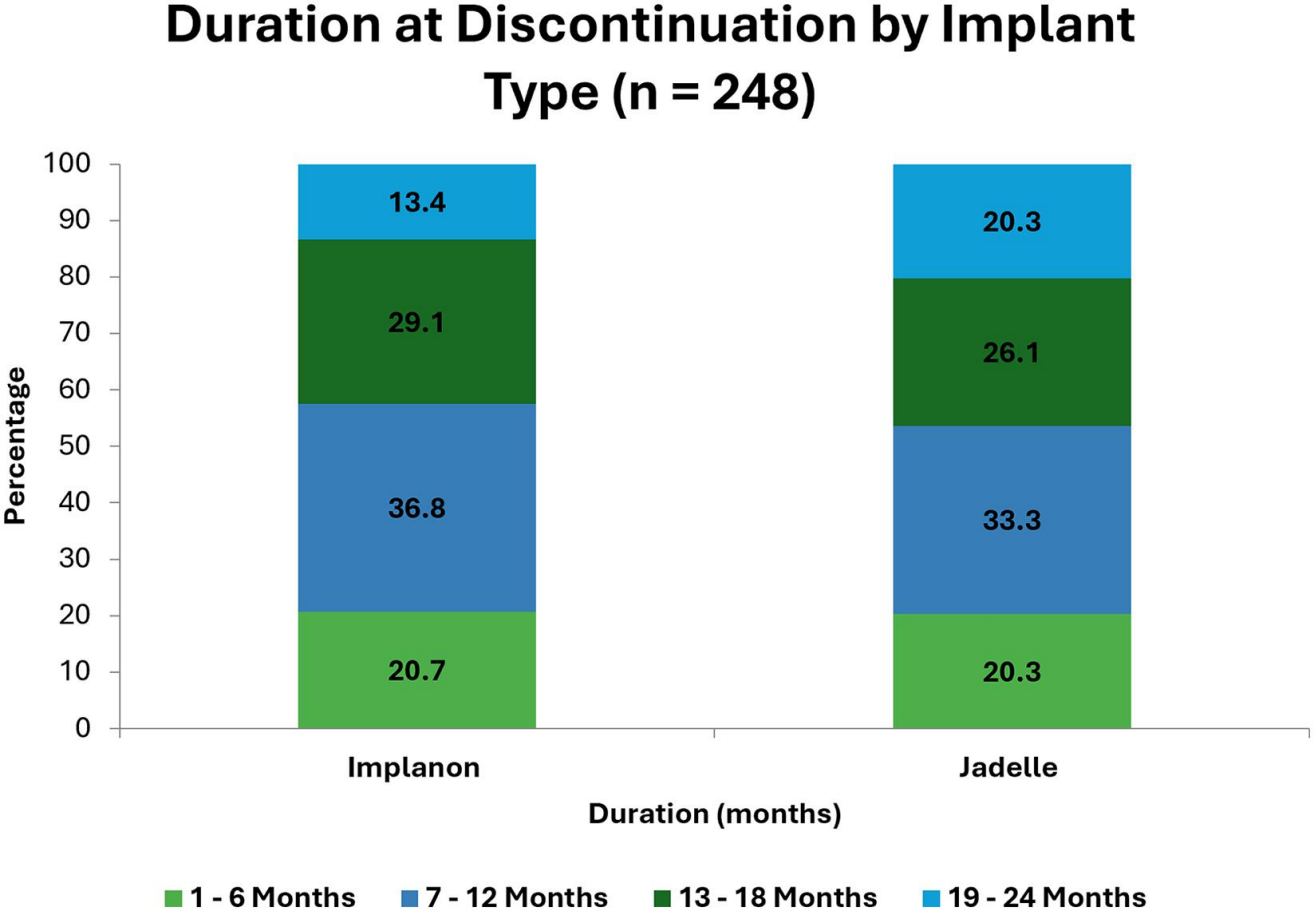
Duration at discontinuation by implant type.

Figure 1 shows the distribution of discontinuation timing. Implanon users had a greater tendency for very early removals (≤12 months) compared to Jadelle (Figure 2).

### Reasons for early implanon discontinuation

**Figure 3:**
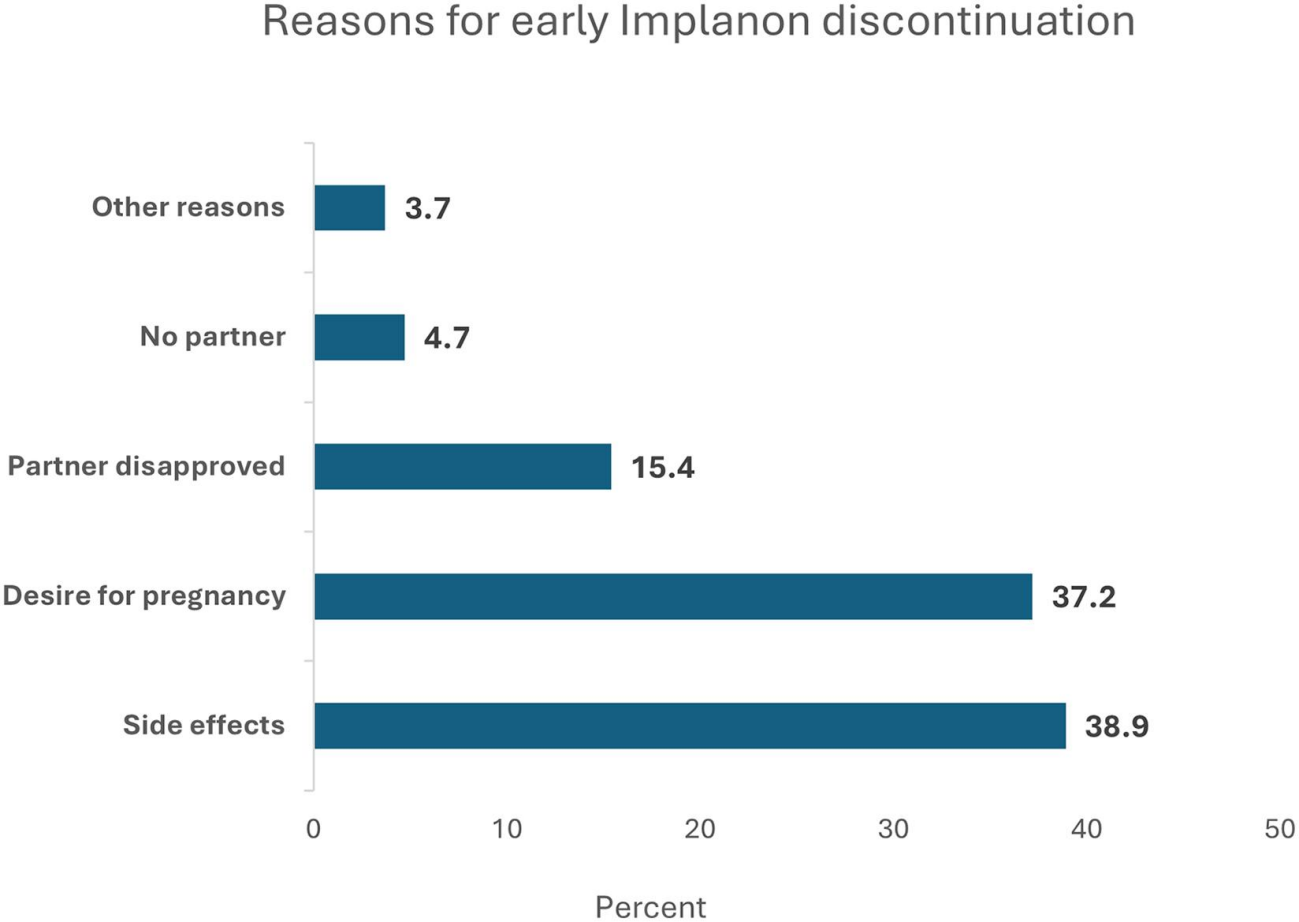
Reasons for early implanon discontinuation.

Figure 3 reported reasons for implant removal. The most frequently cited reason was side effects 38.9%, followed closely by desire for pregnancy 37.2%. Partner disapproval accounted for 15.4% and other reasons 3.7% (Menopause, Ageing and medical condition) were less commonly reported.

### Factors associated with early implant discontinuation

Table 4 shows the relationship between factors associated with early implant discontinuation among women of reproductive age. The relationship between each independent variable and early implant discontinuation was first analyzed separately using crude logistic regression. In the bivariate analysis, age group, state of residence, and reasons for removal showed significant associations with early discontinuation at *p* ≤ 0.20. Specifically, women aged 35–39 years had lower odds of early discontinuation compared with those aged 25–29 years (COR = 0.55, 95% CI: 0.34–0.86, *p* = 0.01). State-level differences were also observed: women in Enugu were less likely to discontinue early (COR = 0.47, 95% CI: 0.28–0.79, *p* = 0.004), while women in Lagos had higher odds (COR = 1.64, 95% CI: 1.11–2.44, *p* = 0.01) compared with Kaduna. In terms of reasons for removal, citing partner disapproval (COR = 2.67, 95% CI: 1.48–4.82, *p* = 0.001) and side effects (COR = 3.45, 95% CI: 2.19–5.42, *p* < 0.001) were strongly associated with early discontinuation compared with desiring pregnancy. The place of implant insertion was associated with early discontinuation. Compared to women who received implants at Primary Health Centers (PHCs), those who obtained their implants from Proprietary Patent Medicine Vendors (PPMVs) had significantly higher odds of early discontinuation (COR = 1.48, 95% CI: 1.06–2.06, *p* = 0.021). Other predictors such as type of implant, and other reasons did not show statistically significant associations.

**Table 4:** Factors associated with early implant discontinuation among women of reproductive Age.

Variable	Category	n	COR (95% CI)	*p*-value	AOR (95% CI)	*p*-value
Age group	15–19 yrs	16	1.38 (0.58–3.27)	0.461	1.11 (0.43–2.84)	0.828
	20–24 yrs	150	1.27 (0.85–1.89)	0.237	1.19 (0.75–1.88)	0.462
	25–29 yrs (ref)	211	1	–	1	–
	30–34 yrs	192	0.88 (0.59–1.32)	0.536	0.83 (0.54–1.27)	0.386
	35–39 yrs	145	0.55 (0.34–0.86)	0.010*	0.50 (0.31–0.80)	0.004*
	40–44 yrs	86	0.92 (0.54–1.56)	0.744	0.86 (0.48–1.52)	0.603
	45 + yrs	74	0.77 (0.42–1.39)	0.382	0.70 (0.37–1.34)	0.283
State of residence	Kaduna (ref)	389	1	–	1	–
	Enugu	61	0.47 (0.28–0.79)	0.004*	0.53 (0.30–0.91)	0.020*
	Kano	195	0.81 (0.55–1.20)	0.289	0.79 (0.51–1.23)	0.298
	Lagos	229	1.64 (1.11–2.44)	0.013*	1.32 (0.85–2.06)	0.214
Place of implant insertion	Pharmacy	9	0.65 (0.23–1.80)	0.402	0.72 (0.26–2.00)	0.523
	PHC (ref)	456	1	–	1	–
	PPMV	346	1.48 (1.06–2.06)	0.021*	1.39 (1.00–1.93)	0.049*
	Private Hospital	27	1.21 (0.65–2.26)	0.543	1.15 (0.58–2.25)	0.683
	Secondary Hospital	36	1.36 (0.74–2.48)	0.321	1.26 (0.68–2.35)	0.465
Type of implant removed	Implanon (ref)	606	1	–	1	–
	Jadelle	268	0.99 (0.73–1.34)	0.928	0.94 (0.68–1.30)	0.706
Reason for removal	Desire for pregnancy (ref)	132	1	–	1	–
	Dissatisfaction with implant method	10	1.84 (0.65–5.22)	0.254	1.43 (0.48–4.30)	0.519
	No partner	15	1.33 (0.55–3.22)	0.52	1.20 (0.44–3.27)	0.72
	Other (specify)	8	1.10 (0.35–3.45)	0.873	1.02 (0.28–3.68)	0.97
	Partner disapproved	57	2.67 (1.48–4.82)	0.001	2.31 (1.25–4.26)	0.007*
	Side effects	137	3.45 (2.19–5.42)	0.00	3.01 (1.89–4.78)	0.00

COR, crude odds ratio; AOR, adjusted odds ratio; CI, confidence interval; (ref), reference category.

*p < 0.05.

*p < 0.001.

Multivariable logistic regression was performed to control for confounding variables and to identify independent predictors of early implant discontinuation. After adjustment, maternal age, state of residence, place of implant insertion, and reasons for removal remained significant predictors. Women aged 35–39 years continued to show reduced odds of early discontinuation compared with those aged 25–29 years (AOR = 0.50, 95% CI: 0.31–0.80, *p* = 0.004). Women in Enugu had significantly lower odds of early discontinuation compared with those in Kaduna (AOR = 0.53, 95% CI: 0.30–0.91, *p* = 0.02). Regarding reasons for removal, women who discontinued due to partner disapproval (AOR = 2.31, 95% CI: 1.25–4.26, *p* = 0.007) and those citing side effects (AOR = 3.01, 95% CI: 1.89–4.78, *p* < 0.001) had substantially higher odds of early discontinuation compared with those that did not mention side effects as reasons for early implant removal. The association between place of implant insertion and early discontinuation persisted for PPMVs. Women who received implants from PPMVs remained significantly more likely to discontinue early compared to those who received services at PHCs (AOR = 1.39, 95% CI: 1.00–1.93, *p* = 0.049).

## Discussion

### Discontinuation rate by type of implant

This study examined the factors influencing early discontinuation of contraceptive implants among women accessing services from trained community-based health providers across four Nigerian states. First, among women who removed implants (874), the percentage of early implant withdrawal (248) was 28.4%. This result is consistent, however higher, with previous studies carried out in two tertiary centers in Ilorin, Nigeria (26.1%) (27) and Enugu, Nigeria (19.3%) (28). The discrepancy may be due to types of clients visiting CPs and PPMVs and the two tertiary institutions in terms of likely higher levels of education and economic status. Furthermore, a research conducted in a medical facility in Uganda's Wakiso district found a 31% early removal rate (29, 30).We found in this study that implanon (29.5%) and Jadelle (25.7) had comparable early discontinuation rates among discontinuers. However, compared to Jadelle users (53.6%), Implanon users were more likely to stop within the first 12 months (57.6% of discontinuers), indicating potential method-specific variations in continuation patterns. This is consistent with a research conducted in Nigeria among patients who came to the FP unit of a tertiary institution in Northeastern Nigeria for implant removal, which discovered that implanon accounted for more than half (53.4%) of all implants removed during the study period (31).

### Age, a key predictor of discontinuation

Secondly, the study revealed age group as a key predictor of discontinuation. In the bivariate analysis, women between the ages of 35 and 39 were 45% less likely to discontinue early than women between the ages of 25 and 29 (COR = 0.55, 95% CI: 0.34–0.86, *p* = 0.01). The multivariate model maintained this protective effect (AOR = 0.50, 95% CI: 0.31–0.80, *p* = 0.004). Older women are less likely to stop using contraceptives, which is consistent with prior research showing that they frequently have more stable fertility choices and a higher tolerance for adverse effects (2–4). While some research indicates older women may be less likely to stop using the implant, age has a mixed effect on early implant cessation. Younger teenagers may be less likely to stop than adults, and other factors include adverse effects,inadequate counseling and the intended length of usage are frequently more powerful indicators. One study, for example, discovered that younger age groups (20–29 years) were more likely to stop than older groups (35 + years) (32). Another study, however, found no discernible age-related variation in early discontinuation (33). Higher rates of early cessation are consistently associated with other characteristics such suffering side effects, not receiving counseling, and planning to use the implant for a shorter period of time (26).

### Geographical disparities in implant continuation

Thirdly, geographical variation was also noted in the study. Women in Lagos had greater odds in the crude model (COR = 1.64, 95 percent CI: 1.11–2.44, *p* = 0.01), but those in Enugu were less likely to stop early than those in Kaduna (AOR = 0.53, 95 percent CI: 0.30–0.91, *p* = 0.02). These variations imply that continuation outcomes may be impacted by state-specific service delivery contexts, such as partner involvement, counseling quality, and follow-up access. Patients in geographically remote areas may have limited access to timely removal services or specialized care for complications, which can result in delayed intervention or unresolved issues, potentially leading to early implant failure. Another way that geography may affect early implant removal are through differences in access to quality healthcare, socioeconomic factors, and potentially environmental influences on general health.

### Side effects: the leading driver of early removal

Fourthly, this study demonstrated that side effects had a major impact on early implant termination. According to the data, women who stopped due to side effects made up 38.9% of removals and were three times more likely to quit early than those who did not (AOR = 3.01, 95 percent CI: 1.89–4.78, *p* < 0.001). Previous studies found that mothers who experienced side effects from Implanon were more likely to stop taking the drug early than mothers who did not (34–42). This is in line with a worldwide trend, and women may find certain side effects intolerable or worry about unjustified issues or myths related to these side effects (9). Inadequate counseling before implantation may also affect a woman's response to possible side effects, which could result in an early removal. In order to identify, evaluate, and manage women who are experiencing side effects, follow-up community healthcare workers could monitor women during the post-insertion period.

### Partner influence: a critical factor

Lastly, we found that partner dissatisfaction (15.4% of removals) was also independently associated with higher probabilities of early discontinuation (AOR = 2.31, 95 percent CI: 1.25–4.26, *p* = 0.007). Partner disapproval is one of the primary reasons contraceptive implants are removed and terminated early. Because of her partner's lack of support or outright rejection, the woman is often under pressure to remove the implant; in some cases, this may involve violence or reproductive coercion (43, 44). Women who resort to not consulting their partners before seeking family planning services may encounter resistance because it goes against the cultural belief that men are the primary decision-makers in the home. Furthermore, it's probable that a large number of men do not engage in pre-insertion counseling before their partners receive contraceptive implants, and they might exert pressure on them to halt the procedure early if they experience adverse effects.

### Place of implant insertion, a key predictor of early discontinuation

Early discontinuation was significantly higher among women who received implants from PPMVs compared to PHCs, even after adjustment (AOR = 1.39, 95% CI: 1.00–1.93, *p* = 0.049), suggesting that differences in service quality and continuity of care may underlie this pattern. Evidence shows that contraceptive discontinuation is often a marker of health system performance, particularly reflecting gaps in counseling, side-effect management, and follow-up support (30, 33). PHCs are generally better integrated into formal systems with structured supervision and client follow-up, whereas PPMVs may have variable adherence to national guidelines and limited capacity for comprehensive counseling and referral. Effective, structured contraceptive counseling has been shown to improve continuation and method satisfaction. Strengthening training, supervision, and linkage between PPMVs and PHCs is therefore critical to sustaining both access and quality of implant services (45).

### Implications

Overall, these findings emphasize that beyond fertility intentions, side-effect management and male partner influence remain critical determinants of implant continuation. This echo results from other African studies documenting high rates of discontinuation linked to side effects and partner opposition (5, 7, 8, 11). Early removal due to side effects reflects client dissatisfaction and reduces confidence in services, potentially affecting broader contraceptive uptake. This suggests the need for relevant stakeholders and trainers to equip CPs and PPMVs with basic management guidelines for common side effects and referral pathways. Male partner influence significantly drives early discontinuation, reflecting gender power dynamics and undermines continuation. Therefore, implementing partners and government stakeholders including men in family planning education and counseling sessions is one suggested strategy to reduce resistance, dispel misconceptions, and promote cooperative decision-making for better contraceptive outcomes. Likewise, the findings reinforce the role of community-based providers such as pharmacists and PPMVs in expanding access to implants. While they successfully provided insertion and removal services, the results indicate a need for stronger pre-insertion counseling, improved follow-up mechanisms, and targeted male partner engagement strategies, to be implemented by ministries of health at the federal and state levels and implementing partners. Strengthening these areas may help reduce premature discontinuation and enhance women's satisfaction with implant use (1, 8, 17, 21–23).

### Limitations and strengths

This study is not without limitations. First, the study relied on routine service delivery data, certain key variables, including socioeconomic status, parity, and prior contraceptive use, were not available, limiting the ability to fully adjust for confounders. Another limitation is the small sample sizes in some subgroups led to non-estimable odds ratios, and the cross-sectional design restricts causal inference, as associations cannot establish temporality. The third limitation is that this study gave equal weight to pregnancy as a reason for removal as other factors such as side effects and it is not a reflection of poor counseling. Pregnancy was not excluded from the analysis. Despite these limitations, the study drew on a relatively large, multi-state sample of 874 women, providing real-world insights into implant discontinuation across diverse contexts in Nigeria. The inclusion of both Implanon and Jadelle users enabled method-specific comparisons, and the use of multivariate logistic regression strengthened the robustness of the findings.

## Conclusions

This study highlights that one-third of discontinuers did so early at less than 24 months (28.4%), with side effects and partner disapproval being the primary drivers, while older women and residents of Enugu were less likely to discontinue early. These findings emphasize the need for enhanced side-effect counselling, active partner engagement, and strengthened provider capacity to reduce premature removals and improve continuation. By addressing these drivers, Nigeria can optimize the contribution of community-based providers to contraceptive service delivery, enhance client satisfaction, and strengthen progress towards national and global family planning goals.

## Data Availability

The datasets presented in this study can be found in online repositories. The names of the repository/repositories and accession number(s) can be found below: Population Council's Dataverse, https://doi.org/10.7910/DVN/Q0SLQS.
